# Does the histopathological subtype of primary basal cell carcinoma predict the subtype of secondary tumours? What role do genetic mutations play?

**DOI:** 10.1002/ski2.123

**Published:** 2022-05-18

**Authors:** Liam D. Turner, Andrea Zarkovic, Jessica Lee Siew Hua, Weng Chan, Siddharth Ogra, Daniel Brettell, Oded Ohana, Pavindran Gounder, Mark Hayes, Simon Madge

**Affiliations:** ^1^ Department of Ophthalmology Hereford County Hospital Hereford UK

## Abstract

**Background:**

Basal cell carcinoma (BCC) is one of the most common malignancies in the world. The frequency of histopathological subtypes and the distribution on the body of BCC has been well documented. Less has been written on the nature of secondary tumours. The genetics of BCC is starting to be understood, particularly with the advent of newer medical treatments (hedgehog inhibitors).

**Objectives:**

To determine if primary basal cell carcinoma histopathological subtype predicts secondary tumour subtype, as well as their anatomical distribution.

**Methods:**

A retrospective case series of patients over the age of 18 was performed from 2009 to 2014, with at least two separate diagnoses of BCC.

**Results:**

In 394 identified patients, a total of 1355 BCCs arose in the cohort over the 6‐year study period. The number of secondary BCCs per patient ranged from 2 to 19 tumours. Nodular BCC was the most likely to reoccur in secondary tumours (53.3%), followed by mixed subtypes (45.7%).

**Conclusions:**

Within our study, we did find a predisposition for secondary BCCs to be of the same histopathological subtype as the primary, particularly with respect to nodular and mixed tumours. Furthermore, we found that secondary tumours were also more likely to occur on the same anatomical site as the primary tumour. We are only just beginning to under the genetic mutations involved in subtype formation.

1

**What is already known about this topic?**

- The frequency of histopathological subtypes of basal cell carcinoma across different population groups is well documented, with nodular subtypes being the most common irrespective of the group in question. Furthermore, there is a predisposition for tumours to arise on particular regions of the human body. The majority of basal cell carcinomas arise on the head and neck region likely representing the greater ultraviolet radiation exposure risk of this anatomical site.

**What does this study add?**

- This study specifically looks at the histopathological subtypes of secondary basal cell carcinoma (those who have had a primary tumour, not recurrent tumours). It shows that primary tumour subtype can be predictive of secondary tumour subtype, especially with respect to nodular and mixed histopathological subtypes.

**What are the clinical implications of this work?**

- It highlights the higher rates of mixed subtypes in secondary tumours. This is of importance to the clinician as mixed subtype tumours need to be managed more cautiously than nodular tumours. Furthermore, it may assist to further understand the genetics of histopathological subtypes as one might expect that certain mutations may underly the type of basal cell carcinoma.

## 2 INTRODUCTION

Non‐melanoma skin cancer (NMSC) is the most common malignancy in fair complexion populations worldwide and within the top five cancers throughout the world according to the World Health Organisation (WHO).^1^, ^2^, ^3^ Basal cell carcinoma (BCC) accounts for the majority of NMSCs, with rates as high as 800/100 000 population/year in Australia and New Zealand and as low as 1–5/100 000 in African and Asian countries.^4^ This difference is likely attributable to skin pigmentation, societal age and reporting systems. The mortality from BCC is low, less than 1.9/100 000 person‐years at risk, but the morbidity due to potential loss of function, cosmetic disfigurement and cost to society are enormous.^5^

Risk stratification of BCC into either low or high‐risk is determinant upon a number of factors, including: location, tumour size, primary versus recurrent, presence of previous radiotherapy, presence of immunosuppression, perineural invasion and histopathological subtype.^6^, ^7^ Five main histopathological subtypes of BCC exist, including: nodular, superficial, micronodular, infiltrative and morpheaform. The latter three are considered high‐risk, and the former two low‐risk.^7^, ^8^

Treatments for basal cell carcinoma vary, based on the histopathological type, with low‐risk basal cell carcinoma, superficial subtype, amenable to topical therapies (e.g., 5% imiquimoid, 5‐fluorouracil),^9^, ^10^ and local destructive therapies (e.g., cryotherapy).^11^ High risk basal carcinoma requires either surgery, radiotherapy^12^ or oral hedgehog pathway‐inhibitors, for example, vismodegib or sonidegib.^13^, ^14^, ^15^ As a result of the advent of these latter treatments, the underlying genetic basis for BCC is being elucidated. One such driver for BCC development, is the activation of the hedgehog pathway with mutations of Patched1 (PTCH1) gene identified in the majority of tumours.^13^, ^14^, ^16^ Since BCCs display great variability with respect to histopathological features, prognosis and subsequent response to treatment, determining the underlying genetic profile for the varying subtypes may improve directed therapies and prognosis.

At present, the genetic basis for different histopathological subtypes of BCC has not been determined. If BCC subtypes do have differing aetiology, one might expect individuals with multiple tumours to express similar histopathology/morphology within subsequent tumours in the same individual. The purpose of this study was to determine the histopathological subtype of subsequent BCCs in patients with an initial primary BCC, as well as their anatomical distribution.

## 3 MATERIALS AND METHODS

A retrospective analysis of patients over the age of 18 was performed from 2009 to 2014, with at least two separate diagnoses of BCC. The diagnosis was confirmed through accessing the pathology database at Hereford County Hospital, Hereford, United Kingdom. In patients where incisional and excisional biopsies were available for the same lesion, the incisional biopsy result was excluded.

The first biopsy proven BCC was considered the patient's primary BCC, with subsequent BCCs considered secondary. To ensure that only new secondary tumours were included, any tumour occurring at the same site as a previous lesion were considered recurrent, and therefore excluded from analysis. The pathology database prior to 2009 was searched to ensure that the primary BCC was in fact the patient's primary for the purpose of the study. This study was approved by the Local Research Ethics Committee and complies with the Declaration of Helsinki.

Demographic data as well as geographic distribution, histological subtype and timeframe between lesions was collected. In cases of mixed histological sub‐types, each component was recorded. Other histologically proven skin malignancies were also noted. Statistical analysis was carried out using Statistical Package for the Social Sciences 17.0 (IBM SPSS Statistics for Windows, Version 17.0).

## 4 RESULTS

In 394 identified patients, a total of 1355 BCCs arose in the cohort over the 6‐year study period. The mean age at time of initial BCC diagnosis was 70.9 ± 11.3 years. Males accounted for 67.5% of patients. There was no difference in the mean age of men and women at presentation (71.2 ± 10.4 vs. 70.4 ± 12.9 years respectively, *p* = 0.501).

The number of secondary BCCs per patient ranged from 2 to 19 tumours, with 68.7% having 3 or fewer lesions in total (Table 1). Patients with 4 or more BCCs tended to develop their first BCC at an earlier age compared to those patients with 3 or fewer BCCs (68.7 ± 11.2 vs. 72.0 ± 11.2 respectively, *p* < 0.01).

**TABLE 1:** Secondary basal cell carcinoma (BCC) lesions

Number of secondary BCC lesions	Number of patients (*n*, (%))	Male (*n*, (%))	Female (*n*, (%))
2	185 (46.9)	108 (58.3)	77 (41.7)
3	86 (21.8)	53 (61.6)	33 (38.4)
4	46 (11.7)	39 (84.8)	7 (15.2)
5	27 (6.8)	21 (77.7)	6 (22.3)
6	13 (3.3)	11 (78.6)	2 (21.4)
7	13 (3.3)	12 (92.3)	1 (7.7)
8	10 (2.5)	10 (100)	0 (0)
9	3 (0.75)	3 (100)	0 (0)
≥10	11 (2.7)	10 (90.9)	1 (9)
Total	394	267	127

Men had significantly more subsequent BCC lesions than women (3.8 ± 2.6 vs. 2.7 ± 1.3, *p* < 0.001). 86.1% of patients with four or more lesions were men, and significantly more men than women who had subsequent BCC lesions had more than 4 lesions (*p* ≤ 0.001).

The anatomical distribution of initial and subsequent BCCs is presented in Table 2. The head and neck region was involved in 833 cases (61%), followed by the torso (*n* = 359, 26.5%), lower limbs (*n* = 71, 5.2%) and upper limbs (*n* = 68, 5%).

**TABLE 2:** Anatomical distribution of basal cell carcinomas and probability of subsequent lesions to occur in the same anatomical location

	Primary *N*, (%)	Subsequent *N*, (%)	Subsequent lesion in same anatomical location (%)	Subsequent lesion in different anatomical location (%)	Total
Head and neck	251 (65.7)	582 (61.3)	76	24	833
Torso	74 (19.4)	285 (30.0)	52	48	359
Upper limbs	32 (8.4)	36 (3.8)	25	75	68
Lower limbs	25 (6.5)	46 (4.8)	17	83	71
Not recorded					24
Total	382	949			1355

Of the 1355 total lesions, histological subtypes were noted in 1220 cases. Table 3 shows the histological subtypes of primary and secondary BCCs. Nodular BCCs were the most common histological subtype in both primary and secondary BCCs, accounting for approximately 37.3% of primary and secondary tumours. Grouping of histological subtypes by less aggressive (nodular and superficial) versus more aggressive subtypes (micronodular, infiltrative and morphoeic) showed a stable proportion of cases in each group in both primary and secondary BCCs. Patients with more aggressive histological subtypes on their initial BCC lesion were older at first diagnosis, with a mean age of 74.5 ± 9.3 years compared to 70.3 ± 11.6 years for patients with less histologically aggressive tumours (*p* < 0.001). There was no statistical difference with regards to the number of secondary BCCs between the two groups (3.2 ± 2.0 vs. 2.9 ± 1.7 respectively, *p* = 0.11). Lesions with mixed subtypes accounted for 22.7% (*n* = 308) of all lesions. A nodular component was seen in 65.8% of these (*n* = 204). A mixed aggressive component (micronodular infiltrative, morphoeic, or a combination of these) was present in 61% (*n* = 125) of these cases. Secondary BCCs were more likely to be of a mixed histological subtype.

**TABLE 3:** Histological distribution of primary and subsequent basal cell carcinomas

Basal cell carcinoma #	# Patients	Nodular	Superficial	Infiltrative	Micronodular	Morphoeic	Mixed	Other
1	394	140	47	45	14	5	74	69
2	394	149	43	51	15	2	106	28
3	208	81	31	26	9	3	47	11
4	122	50	14	15	4	0	29	10
5	75	22	13	11	4	0	20	5
6	50	24	6	7	5	0	5	3
7	35	13	2	4	1	0	14	1
8	23	9	3	6	0	0	4	1
9	13	4	0	1	0	0	3	5
≥10	41	14	13	3	2	1	6	2

The distribution of the different histological subtypes by anatomical location is presented in Table 4. Nodular BCCs were the most common. Superficial BCC lesions were relatively rare in the head and neck region and accounted for 6.1% of lesions in this area. The likelihood of patients presenting with the same histology subtype in recurrent tumours was stratified by anatomical location as well as primary histological subtype. This is shown in Table 5. The head and neck area as well as torso were most likely to show the same histological subtype for secondary tumours (45.7%, 41.2% respectively). Nodular BCC was the most likely to reoccur in secondary tumours (53.3%), followed by mixed subtypes (45.7%).

**TABLE 4:** Histologic subtypes of basal cell carcinoma lesions by anatomic location

Location		Nodular	Superficial	Infiltrative	Micronodular	Morphoeic	Mixed	Total
Head and neck	*n*	350	46	124	34	8	193	755
	%	46.0	6.1	16.5	4.5	1.0	25.5	
Upper limb	*n*	14	15	11	1	1	18	60
	%	23.0	25.0	18.0	1.7	1.7	30.0	
Torso	*n*	115	97	27	15	2	84	340
	%	33.9	28.5	8.0	4.4	0.6	24.7	
Lower limb	*n*	27	14	7	4	0	13	65
	%	41.5	21.5	10.8	6.2	0.0	20.0	
Total	*n*	506	172	169	54	11	308	1220

**TABLE 5:** Probability of a subsequent primary basal cell carcinoma (BCC) displaying the same histological features as the previous lesions according to the anatomical location (a) and histopathological subtype (b)

	(a) Anatomical location of BCC			
	Head and neck	Torso	Upper limb	Lower limb
Probability of same histological subtype	45.7%	41.2%	31.6%	25%

	(b) Histological subtype of BCC					
	Nodular	Superficial	Infiltrative	Micronodular	Morphoeic	Mixed
Probability of same histological subtype	53.3%	32.6%	30.8%	11.1%	0%	45.7%

The mean time interval between primary and secondary BCCs occurrence was 28.0 ± 41.36 months (median 12 months). Twenty percent of patients developed a subsequent BCC within a year of initial diagnosis. A linear regression model found the time interval between BCC occurrences to be inversely proportional to the number of subsequent BCCs. Patients with a greater number of BCCs tended to have a shorter interval between subsequent BCCs (*p* < 0.001).

## 5 DISCUSSION

The risk of secondary tumours in patients with a primary basal cell carcinoma has been well documented in the literature.^17^ A recent meta‐analysis showed that there was approximately a 30% risk of developing a secondary tumour after an initial diagnosis of BCC and that the risk increased per year of follow‐up.^18^ Individuals with an initial tumour have a 10‐fold increased risk of further BCC compared to the general population.^19^ However, an understanding of the nature of secondary tumours, in terms of histopathological subtype and anatomical location, has not been well‐documented. It was hypothesised that if histopathological subtypes do have an underlying genetic aetiology, then it would be expected that secondary tumours would be of similar subtype.

Nodular BCC was the most common histopathological subtype within our study at around 38%, followed by mixed at 25% and superficial at 13%, with the high‐risk subtypes (infiltrative, micronodular and morpheoic) all occurring less frequently. This frequency of tumour types is fairly consistent within the existing literature, with nodular and superficial BCC being predominant across nearly all studies, with rates ranging from 39.5% to 78.7% for nodular and 3%–43% for superficial, dependent on the patient cohort in question.^20^, ^21^, ^22^ It has been suggested that rates of superficial BCC may be under reported due to the tendency for these lesions to be treated with topical treatment as opposed to surgical intervention with formal histopathology.^15^ Furthermore, rates of high‐risk tumour types (i.e., infiltrative, micronodular, morpheaform) are often difficult to determine as much of the literature combines them together in order to simplify classification.^23^, ^24^

The probability of secondary tumours having the same histopathology as their primary tumour were high, at approximately 0.53 (53%) for nodular and 0.45 (45%) for mixed BCC. In other words, a patient who presents with a nodular or mixed basal cell carcinoma as their primary tumour have a one in two chance of their secondary tumours being of the same histopathological subtype. Does this represent an underlying genetic predisposition within an individual or is it a result of a common risk factor profile, or is it a combination of both? At the genetic level, the main driver for BCC formation is the activation of the hedgehog pathway (Hg). PTCH1 is a tumour suppressor gene and inactivating mutations of it lead to unregulated cellular differentiation. Inactivating mutations of PTCH1 and SMO, occur in 90% and 10%, respectively of basal cell carcinoma.^16^ Somatic mutations arise due to the effects of ultraviolet radiation to DNA within basaloid keratinocytes.

Recent publications have tried to elucidate the genetic profiles of BCC subtypes.^16^, ^25^, ^26^, ^27^ There has been a strong association identified between expression of the PTCH1 gene in low‐risk tumours (i.e., nodular and superficial) compared to high‐risk tumours (i.e., micronodular and infiltrative), although this was not found to be statistically significant.^16^ A more recent study looked specifically at low‐risk BCCs including nodular and superficial variants and found that both the NOTCH1 and PTCH1 gene were more frequent in superficial than nodular BCCs.^28^ They also noted that PTCH1 mutation was significantly associated with intermittent sun exposure and the development of a single BCC, which would be consistent with nodular and superficial subtypes being the most common.^28^ Furthermore, NOTCH1‐mutation lesions were more likely to be located on the trunk. This is in line with the fact that superficial BCCs are more commonly found on the trunk and are believed to result from more intermittent and intense sun exposure.

Additionally, a study by Rao et al.^29^ found that further genetic associations characterised by the expression of EZH2 and Ki67 correlated with aggressive subtypes. In comparison with less aggressive subtypes, H3K27mc3 and 5 hmC upregulation positively correlated with the occurrence of less aggressive histopathology.^29^ Finally, there is a suggestion that basosquamous tumours, a high‐risk subvariant of BCC, are less chromosomally stable than lower risk BCC. Basosquamous tumours have a greater number of chromosomal alterations, including amplification of chromosome 6, which may be associated with its increased metastatic potential.^27^

In terms of anatomical location, secondary BCCs are more likely to arise in the same region of the body as their primary tumour. The probability of developing a secondary tumour at the same anatomical site was almost double for tumours occurring on the torso, upper and lower limbs with rates of 52%, 25% and 17%, respectively. The appearance of a basal cell carcinoma in a specific location likely represents a culmination of a number of factors, including but not limited to, genetic predisposition, sun exposure, skin type, to name a few. Furthermore, it is also possible that there are inherent features of certain BCC lesions that predispose their development in certain anatomical locations.^15^

Gender was also found to be an important risk factor for secondary BCC's, with greater than 80% of patients with four or more tumours being of male gender. Male gender is a known independent risk factor for BCC development.^30^, ^31^ Males are more commonly affected by BCC than women, except in the younger age groups (<40 years of age).^32^ This propensity for males to acquire BCC more frequently than females is likely multifactorial. Males are exposed to a greater degree of occupational ultraviolet radiation, and less likely to engage in sun protective practices (i.e., sunscreen application, hat and sunglasses wear, avoiding midday sun).^33^, ^34^ Some studies in mice models have even suggested a potential genetic basis, with male mice having lower cutaneous antioxidants and increased oxidative damage to DNA, as a result of ultraviolet radiation exposure comparatively to female mice.^35^

At present, the aetiology of different histopathological subtypes of BCC is not known, with further research required. Within our study, we did find a predisposition for secondary BCCs to be of the same histopathological subtype as the primary, particularly with respect to nodular and mixed tumours, with 53.3% and 45.7% probability, respectively. Furthermore, we found that secondary tumours were also more likely to occur on the same anatomical site as the primary tumour. In terms of human cancers, BCC have one of the greatest number of associated mutations.^36^ Therefore, it is likely that numerous genes are involved in the determination of the subtype of a BCC. These genes are only just starting to be determined and uncovered. The determination of the genetic basis of BCC histopathological subtypes will ultimately assist clinicians in their treatment of patients, with the development of further targeted therapies.

## AUTHOR CONTRIBUTIONS

**Liam D. Turner:** Conceptualization (lead); Resources (lead); Writing – original draft (lead); Writing – review & editing (lead). **Andrea Zarkovic:** Conceptualization (lead); Data curation (lead); Formal analysis (equal); Investigation (equal); Methodology (equal); Project administration (equal). **Jessica Lee Siew Hua:** Conceptualization (equal); Data curation (equal); Formal analysis (equal); Investigation (equal); Methodology (equal). **Weng Chan:** Data curation (lead); Formal analysis (lead). **Siddharth Ogra:** Data curation (equal); Formal analysis (equal). **Daniel Brettell:** Data curation (equal); Formal analysis (equal). **Oded Ohana:** Formal analysis (equal); Investigation (equal); Methodology (equal). **Pavindran Gounder:** Data curation (equal); Formal analysis (equal). **Mark Hayes:** Conceptualization (equal); Investigation (equal). **Simon Madge:** Conceptualization (lead); Data curation (equal); Project administration (lead); Supervision (lead); Writing – review & editing (equal).

## CONFLICTS OF INTEREST

None to declare.

## ETHICS STATEMENT

This study was approved by the Local Research Ethics Committee and complies with the Declaration of Helsinki.

## Data Availability

Data available on request due to privacy/ethical restrictions.
